# Wilms' tumor: An update

**DOI:** 10.4103/0970-1591.36722

**Published:** 2007

**Authors:** Hemant B. Tongaonkar, Sajid S. Qureshi, Purna A. Kurkure, Mary-Ann A. Muckaden, Brijesh Arora, Thyavihalli B. Yuvaraja

**Affiliations:** Department of Surgical Oncology, Urologic Oncology Service and Paediatric Oncology Service, Tata Memorial Hospital, Mumbai, India; * Department of Paediatric Medical Oncology, Tata Memorial Hospital, Mumbai, India; ** Department of Radiation Oncology, Tata Memorial Hospital, Mumbai, India

**Keywords:** Chemotherapy, National Wilms' Tumor Study Group, International Society of Pediatric Oncology, surgery, survival, toxicity, Wilms' tumor

## Abstract

Wilms' tumor (WT) is the commonest pediatric renal tumor, predominantly seen in children less than five years of age. The majority of patients present with an abdominal lump and CT scan is the usual imaging modality for determining the extent of disease. With multimodality management, the results of treatment of WT have improved dramatically over the last 50 years. The treatment protocols have been devised and modified repeatedly depending on evidence from randomized trials by several cooperative groups - mainly National Wilms' Tumor Study Group (NWTSG) and the International Society of Pediatric Oncology (SIOP). The NWTSG recommends primary surgery followed by chemotherapy while SIOP advocates four weeks of chemotherapy prior to surgery. The regimen, dose and duration of chemotherapy have been repeatedly modified to reduce toxicity while maintaining efficacy. The role of radiation therapy has also been customized. Most centers have reported excellent survival rates with the modern day treatment protocols, except in patients with an unfavorable histology. The results of treatment of relapsed WT have also improved with newer drugs and combinations being used for the same.

While reviewing the existing literature on childhood renal tumors in 1899, Max Wilms, a surgeon, realized the presence of common features in lesions described by different names such as congenital sarcoma of kidney, embryonal sarcoma, adenomyosarcoma, mesoblastic sarcoma and sarcoma muscularis. Wilms added seven cases of his own and provided the classic description of a tumor, which is known today by his name and is one of the commonest renal tumors in children.[1]

The treatment of Wilms' tumor (WT) has evolved from surgical excision as the prime method of treatment to combined multimodal treatment. In 1877, Jessop in Leeds, England performed the first nephrectomy for WT in a two-year-old child.[2] This successful hallmark operation is also noteworthy in that it was reported in Lancet a scant nine days later. The patient succumbed to recurrence nine months after the operation. William Ladd, the father of American Pediatric surgery, standardized the surgical therapy by refining operative technique, with a resultant decrease in surgical mortality.

Contemporary treatment protocols have led to an overall survival of 85% in WT patients and with this, the emphasis has now shifted to individualization of treatment with the aim of reducing the morbidity of treatment without affecting survival. Large randomized controlled trials by various collaborative groups, including the National Wilms Tumor Study Group (NWTSG), The International Society of Pediatric Oncology (SIOP) and the United Kingdom Children's Cancer Study Group (UKCCSG) have facilitated WT treatment to be customized to minimize morbidity for low-risk disease and to maximize the oncologic outcome for high-stage, high-risk patients. Consequently, the outcome for patients with WT has improved remarkably in the past few decades.

## METHODOLOGY

In this article, we review the literature regarding the development of management protocols for WT, based on major randomized clinical trials reported from large cooperative groups; and define the current standard of care and advances made in the management of WT.

## RISK FACTORS

Wilms' tumor is the commonest childhood renal tumor and accounts for 6% of all pediatric malignancies.[3] It predominantly affects children less than five years of age, with 90% of new cases diagnosed before age three years. Occasionally, WT has also been described in teenagers and adults.

Wilms' tumor normally develops in otherwise healthy children; however, 10% of cases occur in individuals with recognizable phenotypic syndromes - either overgrowth or non-overgrowth.[4] The commonest syndromes associated with WT are WAGR syndrome, the Beckwith-Wiedermann syndrome and the Denys-Drash syndrome.

Several epidemiological studies have investigated parental occupational, environmental and lifestyle characteristics as well as birth weight of the child as potential risk factors for Wilms' tumor, but findings to date have been inconsistent and have not been consistently replicated in multiple, high-quality studies in different populations.[5,6] Future epidemiologic studies may benefit from more detailed exposure assessment, validated by environmental and biologic measurements.

## CYTOGENETICS

Wilms' tumor is predominantly a sporadic disease. Genetic predisposition, however, has been demonstrated in a few patients. Substantial bodies of genetic and molecular studies have contributed important insights into understanding the pathogenesis of WT with several genes being implicated in its etiopathogenesis.[7,8]

The WT gene-1 (WT1) is a tumor suppressor gene located on the short arm of Chromosome 11 (11p13). The normal function of WT1 is required for normal genitourinary development and is important for differentiation of the renal blastema. The identification of this suppressor gene was made on cytogenetic analysis of patients with WAGR syndrome who have more than 30% risk of developing WT. A gene that causes aniridia (PAX-6) is located near the WT1 gene on Chromosome 11p13 and deletions encompassing the WT1 and aniridia genes explain the association between aniridia and WT. The Denys-Drash syndrome has a 95% chance of WT development and is another syndrome associated with WT1 gene mutation. Although WT1 has a clear role in the tumorigenesis of WT in the above patients, only a small number of patients with sporadic WT have WT1 mutations suggesting that other genes are involved in WT development. A second WT suppressor gene, WT2, was identified at Chromosome 11p15. Patients with Beckwith-Wiedemann syndrome (BWS) have gene locus in this region and have a 5% risk of developing WT. In addition to the two genetic loci on Chromosome 11, familial WT predisposition at FWT 1 (17q) and FWT 2 (19q) loci has been identified. Several other genetic loci have been implicated in WT by Loss of heterozygosity (LOH) studies or by the presence of germline translocations, including 16q, 7p, 11q, 22q and loss of 4q.

## CLINICAL PRESENTATION

There are no specific clinical features of WT. Most commonly patients present with a palpable abdominal mass accidentally noted by the parents or in the course of a routine clinical examination. However, about one-third of patients present with abdominal pain, anorexia, vomiting, malaise or a combination of these symptoms. Gross or microscopic hematuria is found in 30% of patients. In rare cases of renal vein or caval extension of tumor, varicocele, hepatomegaly, ascites or congestive heart failure may be present. Hypertension is present in about 25% and is attributed to increase in renin activity. Occasional presentation in a subset of patients is rapid enlargement of the abdomen associated with fever, anemia and hypertension as a result of sudden subcapsular hemorrhage. In rare cases of renal vein or caval extension of tumor, varicocele, hepatomegaly, ascites or congestive heart failure may be present. Acquired von Willebrand's disease may occur in less than 10% of patients. Features of congenital syndromes associated with WT like genitourinary malformation (hypospadias, cryptorchidism etc), aniridia, BWS-associated facial dysmorphism, hemihypertrophy etc may be present in 13-28% of patients.

## IMAGING

Although most patients undergo Ultrasonography (US) as the initial imaging study, the conventional imaging modality for WT has been a computed tomography (CT) scan. It ascertains features of the renal mass, the extent, status and function of the contralateral kidney and intravascular extension of tumor. Real-time ultrasonography can identify the patency and presence of tumor thrombus in the renal vein and the inferior vena cava. The value of MRI in this disorder is yet to be established, however, a recent study indicated contrast-enhanced CT and T1-W MR images to be of similar potential and superior to US in the diagnosis of nephroblastomatosis and due to the significant radiation dose of serial CT, MR imaging should be the method of choice wherever it is available.[9]

The role of CT scan in the evaluation and subsequent management of pulmonary lesion found only on chest CT scan is controversial and its prognostic importance is equivocal.[10,11] A recent review from National Wilms' Tumor Study (NWTS) 5 of children who had CT-only lung disease demonstrated an inferior outcome for patients treated with vincristine and dactinomycin (two-drug therapy) only, with or without pulmonary radiation therapy (RT), compared with those who received doxorubicin (DOX) in addition to vincristine and dactinomycin (three-drug therapy).[12] In addition, there appeared to be no beneficial effect from lung irradiation on the outcome of CT-only patients when chemotherapy was considered. Another study demonstrated CT-only lesions are not invariably tumor, demonstrating the need for histopathological confirmation.[13]

## STAGING

Due to the different treatment schedules adopted by the two large cooperative study groups, two major staging systems are currently used: a forthright, surgery-based system developed by the NWTSG [Table 1] and a delayed surgery-based system developed by SIOP [Tables 2 and 3]. Although a direct comparison is not practical due to the difference in surgical timing, both staging systems are valuable in predicting outcomes.

**Table 1:** Staging system of the National Wilms' Tumor Study Group (upfront surgery)[14]

Stage 1	Tumor limited to the kidney and completely excised	
	(a)	The tumor was not ruptured before or during removal
	(b)	The vessels of the renal sinus are not involved beyond 2mm
	(c)	There is no residual tumor apparent beyond the margins of excision
Stage II		Tumor extends beyond the kidney but is completely excised
	(a)	No residual tumor is apparent at or beyond the margins of excision
	(b)	Tumor thrombus in vessels outside the kidney is Stage II if the thrombus is removed en bloc with the tumor
		Although tumor biopsy or local spillages confined to the flank were considered Stage II by the NWTSG in the past, such events will be considered Stage III in upcoming COG studies.
Stage III		Residual tumor confined to the abdomen:
	(a)	Lymph nodes in the renal hilum, the periaortic chains or beyond are found to contain tumor
	(b)	Diffuse peritoneal contamination by the tumor
	(c)	Implants are found on the peritoneal surfaces
	(d)	Tumor extends beyond the surgical margins either microscopically or grossly
	(e)	Tumor is not completely resectable because of local infiltration into vital structures
Stage IV		Presence of hematogenous metastases or metastases to distant lymph nodes
Stage V		Bilateral renal involvement at the time of initial diagnosis

**Table 2:** Staging system of SIOP (upfront chemotherapy)[14]

Stage 1		Tumor is limited to kidney or surrounded with fibrous pseudo capsule if outside of the normal contours of the kidney, the renal capsule or pseudo capsule may be infiltrated with the tumor, but it does not reach the outer surface and is completely resected (resection margins ‘clear”)
	(a)	The tumor may be protruding into the pelvic system and “dipping” into the ureter (but it is not infiltrating their walls)
	(b)	The vessels of the renal sinus are not involved
	(c)	Intrarenal vessel involvement may be present
		FNAC or core needle biopsy does not upstage the tumor. The presence of necrotic tumor or chemotherapy-induced changes in the renal/sinus fat and/or outside of the kidney should not be regarded as reason for upstaging a tumor.
Stage II		Tumor extends beyond the kidney but is completely excised or penetrates through the renal capsule and/or fibrous pseudo capsule into perirenal fat but is completely resected (resection margins “clear”)
	(a)	The tumor infiltrates the renal sinus and/or invades blood and lymphatic vessels outside the renal parenchyma but is completely resected
	(b)	The tumor infiltrates adjacent organs or vena cava but is completely resected
Stage III	(a)	Incomplete excision of the tumor which extends beyond resection margins (gross or microscopic tumor remains postoperatively)
	(b)	Any abdominal lymph nodes are involved
	(c)	Tumor rupture before or intraoperatively (irrespective of other criteria for staging)
	(d)	The tumor has penetrated through the peritoneal surface
	(e)	Tumor implants are found on the peritoneal surface
	(f)	The tumor thrombi present at resection margins of vessels or ureter, transected or removed piecemeal by surgeon
	(g)	The tumor has been surgically biopsied (wedge biopsy) prior to preoperative chemotherapy or surgery
		The presence of necrotic tumor or chemotherapy-induced changes in a lymph node or at the resection margins should be regarded as Stage III.
Stage IV		Hematogenous metastases (lung, liver, bone, brain, etc.) or lymph node metastases outside the abdomino-pelvic region
Stage V		Bilateral renal tumors at diagnosis
		Each side should be substaged according to the above criteria

**Table 3:** Stage-wise survival for favorable histology Wilms' tumor patients

	Stage 1	Stage II	Stage III	Stage IV
NWTS-3[30]	92.5% RFS	89.6% RFS	80.4% RFS	76.5% RFS
	97.6% OS at 16 years	92.9% OS at 16 years	86.2% OS at 16 years	79.5% OS at 16 years
NWTS-4[39]	–	83.6% RFS	88.9% RFS	80.6% RFS
	93.8% OS at 8 years	93% OS at 8 years	89.5% OS at 2 years	
NWTS-5[35]	92.4% RFS	–	–	–
	98.3% OS at 4 years			
SIOP 93-01[36]	88.3% RFS	–	–	–
	97% OS at 5 years			
SIOP-9[32]	--	85% RFS	71% RFS	83% 4RFS at 4 years
	88% OS at 2 years (node -ve)	85% OS at 2 years		
UKWG 2-3[37–38]	86.5% EFS	--	--	--
	94.7% OS at 4 years			

## PATHOLOGY

A classic WT is triphasic, with variable proportions of blastemal, stromal and epithelial components. Some WT are monophasic and have a highly aggressive biological behavior. Histological features in the nephrectomy specimen provide important prognostic information for planning treatment. Presence of nuclear atypia, focal/diffuse anaplasia and sarcomatous elements indicate an unfavorable histology, seen in about 5% of all WT.[14] These account for nearly half of all deaths from this disease. Anaplasia is a marker of resistance to chemotherapy but whether it actually signifies aggressiveness in unknown.

The SIOP trials recognized three prognostic groups of renal tumors of childhood: low-risk, intermediate-risk and high-risk tumors.[15]

It is important to look for nephrogenic rests in the nephrectomy specimen of WT. A nephrogenic rest is defined as the persistence of metanephric tissues in the kidney after the 36^th^ week of gestation. As they are found in 30-40% of the kidneys removed for WT they may be considered as precursor of WT.[16] Nephrogenic rests are subclassified by their position within the renal lobe as perilobar or intralobar. The presence of multiple nephrogenic rests is termed as nephroblastomatosis. Only a small number develop clonal transformation into WT while the rest involute spontaneously. The presence of nephrogenic rests within a kidney resected for a Wilms' tumor indicates the need for monitoring the contralateral kidney for tumor development, particularly in young infants.[17]

## PROGNOSTIC FACTORS

The tumor stage at diagnosis, histological features (favorable vs. unfavorable, presence of diffuse anaplasia) and patient age are the most important prognostic determinants which impact on treatment selection and oncological outcome.[18] The LOH at chromosome 1p and 16q was prospectively analyzed by NWTS-5.[19] Tumor-specific LOH for both chromosomes was found in approximately 5% of patients with FH WT and was associated with increased risk of relapse and death.

## TREATMENT

Wilms' tumor can be considered a model for successful multidisciplinary management of cancer, with improvement in survival from a mere 30% in the 1930s to more than 85% at present. It is also an ideal example wherein the treatment protocols have been devised and modified repeatedly depending on evidence emerging from randomized trials conducted by several cooperative groups and individual institutions. The most important contributions have been from NWTSG and SIOP, with large numbers of patients enrolled in their studies but with a philosophical difference in their treatment approach. The NWTSG recommends primary surgery before administration of chemotherapy while SIOP advocates administration of four weeks of chemotherapy prior to surgery. The former approach allows accurate documentation of histology and tumor extent prior to chemotherapy and also enables the collection of untreated tumor for biology studies and provides an unadulterated view of the tumor's molecular biology. The latter approach downstages the disease, makes surgery easier and reduces the chances of spillage with consequent reduction in abdominal and distant relapse but carries a risk of non-WT histology being present in the primary tumor. Besides, histologic response to chemotherapy can be assessed postoperatively which provides valuable prognostic information.[14] The NWTSG advocates preoperative chemotherapy only in the presence of WT in a solitary or horseshoe kidney, bilateral tumors, venal caval thrombus above hepatic veins or severely symptomatic lung metastases.[20] Since both these approaches have yielded excellent results, there is still a debate concerning the preferred approach, but individualization of treatment based on tumor size and extent, general condition of the patient and surgeon's experience would probably be needed for best outcome.

### Surgery

The timing of surgery with regards to preoperative therapy has varied between the European and the North American group. Nevertheless surgical resection is an important constituent in the multimodal management of WT. Radical nephrectomy is the standard of care for these patients with resectable tumors. A Tran peritoneal route is preferred to provide adequate exposure for complete staging, which includes inspection for local tumor extension, hilar and regional lymph nodes, liver metastases and peritoneal seedlings. Debates about the exploration of the contralateral kidney at surgery exist but evidence now suggests it can be omitted. Data from the NWTS 4 study showed that omission of routine exploration does not affect the outcome or management of newly diagnosed WT, if adequate preoperative CT or MRI is obtained.[21] Prevention of tumor spillage should be of prime concern as this has a bearing in upstaging the tumor, hence gentle handling and careful removal is mandatory.[22] The IVC and the renal vein should be palpated for the presence of tumor thrombus, which if present (renal vein thrombus seen in about 6% patients) should removed en bloc with the kidney.[23] Generally, WT does not infiltrate the adjoining structures, hence a radical en bloc resection is rarely needed, however, a wedge resection if can be performed safely may help in down-staging the tumor to Stage II. As regards lymph node dissection, sampling of suspicious lymph node is recommended instead of a formal lymph node dissection. An adequate and careful surgical resection is mandatory for optimal treatment outcome since incomplete resection, tumor spillage and omission of lymph node sampling are all reported to be associated with abdominal recurrence. Although surgical complications have significantly reduced, surgical morbidity should not be overlooked. Indeed surgical specialists who primarily treat children can perform these operations with lower surgical morbidity.[22] Tumor spillage and intraperitoneal dissemination increases the risk of intra abdominal relapse but the current data suggests that overall survival is not adversely affected.[22]

Partial nephrectomy in the routine management of WT has not gained popularity. The reasons being most WT are large or centrally located making only less than 5% eligible for partial nephrectomy at presentation and even after preoperative chemotherapy only about 10% would be feasible for a nephron-sparing surgery.[24,25] These surgeries carry a risk of leaving behind nephrogenic rest in addition to other procedure-related complications. Besides, the rate of renal failure in patients with unilateral WT is less than 1%.[26] Hence partial nephrectomy is only recommended for patients with synchronous or metachronous bilateral tumors, tumors in solitary kidneys, renal insufficiency of any etiology and children with risk of multiple neoplasms such as in BWS. Laparoscopic nephrectomy with lymph node sampling has been described in the literature but long-term experience is lacking.[27]

### Chemotherapy

Chemotherapy plays a very important role in the management of WT. The current first line drugs for WT are vincristine, dactinomycin and doxorubicin. The second line drugs for non-responsive or relapsed disease are ifosphamide, etoposide, carboplatin and cyclophosphamide. Large cooperative groups have different chronology for deliverance of chemotherapy, drug combination and duration, which have been refined over successive trials to optimize survival rates while minimizing acute and long-term toxicities. It is indeed noteworthy that despite these differences, the survival results amongst all groups are similar. The SIOP and UKCCSG group has favored the use of preoperative chemotherapy in an attempt to down-stage the tumor, whereas the NWTSG advocates upfront nephrectomy without preoperative therapy in order to precisely identify the tumor stage.

### Radiotherapy

As a result of the NWTSG and SIOP studies the role of surgery has been customized though not eliminated. Radiation was an important treatment modality in preoperative and adjuvant settings in the earlier studies. With subsequent refinement in therapy with an aim of maximizing cure and reducing morbidity, there are now precise indications for adjuvant radiotherapy. The current standard of care includes flank/abdominal irradiation (10·8Gy in six fractions) for Stage III favorable-histology (FH) tumors and Stage II-III diffuse anaplastic WT.[3]

The role of lung irradiation in metastatic disease is unresolved with difference among the groups. The NWTSG continues to administer whole lung irradiation (12Gy in eight fractions) in patients with pulmonary metastases, while the SIOP group advocates omission of radiotherapy for patients whose lung metastases disappear completely after six weeks of prenephrectomy chemotherapy with vincristine, dactinomycin and doxorubicin. The role of pulmonary irradiation in children with pulmonary metastases visible on CT but not chest radiograph is further mystified. Use of conformal radiotherapy as well as IMRT has led to dose escalation without increase in morbidity. Similarly, use of high-dose intraoperative radiation therapy for WT has been reported.[4]

## TUMOR STAGE AND TREATMENT

The histologic grade and stage of the tumor are the most important determinants of outcome in Wilms' tumor. An accurate intraoperative staging is required to assess the requirements for postoperative treatment with chemotherapy or radiotherapy.

### Stage I FH

Stage I WT FH has an excellent prognosis. The NWTSG recommends primary surgery followed by adjuvant two-drug chemotherapy (vincristine and pulse intensive dactinomycin) for 18 weeks based on NWTSG 1-3 trials,[28–30] whereas the SIOP approach uses the same drugs either side of surgery for a total of eight weeks based on several SIOP studies.[31,32] Radiation therapy is not necessary in these patients if they receive adjuvant chemotherapy, as demonstrated by the initial three NWTSG trials[28–30] as well as the SIOP 5 trial.[33]

Surgery without chemotherapy was evaluated in NWTS-5 in a select group of patients with highly favorable features (infants younger than 24 months and whose nephrectomy specimen weighed less than 550g.[34] The study was designed with a stringent stopping rule (interim analysis of RFS ≤90%) and reported 13.5% relapse rate at two years, mandating the closure of the study. Most patients could be successfully salvaged with chemotherapy, however, with a two-year overall survival (OS) rate of 100%. In the light of this, further studies are re-evaluating the role of nephrectomy alone in this highly selected group of patients.

The NWTS-3[30] reported 92.5% RFS and 97.6% OS at 16 years and NWTS- 5[35] reported 92.4% RFS and 98.3% OS at four years for these patients. Similarly the SIOP 93-01[36] has reported a five-year RFS rate of 88.3% and five-year OS rate of 97% in these patients. The UKWG 2-3 reported 86.5% EFS and 94.7% OS at four years[37,38] [Table 3].

### Stage II

Currently, the NWTS-5[34,35] recommends primary surgery followed by 18 weeks of chemotherapy with vincristine and pulse-intensive dactinomycin. Addition of postoperative radiotherapy (RT) or doxorubicin was not shown to impart survival benefit in the NWTS-3.[30] The SIOP 93-01 trial[36] recommends preoperative four weeks of chemotherapy with vincristine and pulse-intensive dactinomycin and addition of doxorubicin in postoperative chemotherapy for 27 weeks. Node-negative patients do not receive postoperative RT provided they receive postoperative epirubicin (SIOP-9)[32] while node-positive receive 15Gy RT to the tumor bed in addition to three-drug chemotherapy.

The NWTS -3 reported 89.6% RFS and 92.9% OS at 16 years and the NWTS- 4[39] trial 83.6% RFS and 93.8% OS at eight years for Stage II FH WT. The SIOP-9 reported 85% RFS and 88% OS at two years in node-negative stage patients [Table 3].

### Stage III

The NWTS-5 recommends surgery followed by abdominal radiation (10.8Gy) and 24 weeks of chemotherapy with vincristine, doxorubicin and pulse-intensive dactinomycin. The SIOP 93-01 trial recommends preoperative four weeks of chemotherapy with vincristine and pulse-intensive dactinomycin and 27 weeks of three-drug chemotherapy with vincristine, doxorubicin and dactinomycin in addition to post-perative 15Gy abdominal radiotherapy.

The results of the NWTS-3 trial showed that the addition of doxorubicin to chemotherapy resulted in reduction of radiation dose from 20Gy to 10Gy for Stage III/FH patients. The NWTS-4 trial reported six months chemotherapy to be sufficient for Stage III/FH patients.

The NWTS-3 has reported 80.4% RFS and 86.2% OS at 16 years and NWTS- 4 reported 88.9% RFS and 93% OS at eight years for Stage III FH patients. The SIOP-9 reported 71% RFS and 85% OS at two years in patients with Stage III and Stage II node-positive FH patients [Table 3].

### Stage IV

The NWTSG recommends nephrectomy with lymph node sampling, abdominal radiation 10.8Gy according to local stage of renal tumor (i.e. for Stage III), bilateral pulmonary radiation 12Gy for patients with chest X-ray evidence of pulmonary metastases and 24 weeks of chemotherapy with vincristine, doxorubicin and pulse-intensive dactinomycin. The NWTS-3 reported 76.5% RFS and 79.5% OS at 16 years and NWTS- 4 reported two-year RFS of 80.6% and OS of 89.5% for Stage IV FH patients.

The SIOP advocates six weeks of preoperative chemotherapy with vincristine, dactinomycin and doxorubicin followed by surgery. Those who attain CR by Week 9 receive 27 weeks of three-drug chemotherapy as the preoperative one without radiation therapy. Those whose pulmonary metastases do not respond completely to chemotherapy, with or without surgical excision of residual metastases by Week 9 are advised postoperative 12Gy whole-lung irradiation and four-drug chemotherapy (ifosphamide, carboplatin, etoposide and doxorubicin) for 37 weeks. This helps in limiting the number of children who are exposed to whole-lung radiation, with its inherent associated toxicity.[40] The SIOP reported 83% four year RFS [Table 3].

## MANAGEMENT OF ANAPLASTIC TUMORS

Stage I: Focal or diffuse anaplastic tumors: The NWTS- 5 trial has reported 69.5% RFS and 82.6% OS at four years for Atage I focal or diffuse anaplastic histology patients, using surgery followed by 18 weeks of chemotherapy with vincristine and pulse-intensive dactinomycin. However, due to these suboptimal results, the future studies plan to include abdominal radiation and doxorubicin into the treatment regimen of these patients. The SIOP approach uses the same drugs either side of nephrectomy for a total of eight weeks.

Stages II-IV: For patients with Stage II-IV WT with focal anaplasia, NWTSG-5 advocates primary surgery followed by abdominal radiation and adjuvant three-drug chemotherapy with vincristine, doxorubicin and dactinomycin. For those with diffuse anaplasia, patients are advised postoperative abdominal radiation and adjuvant chemotherapy regimen consisting of vincristine, doxorubicin and cyclophosphamide alternating with cyclophosphamide and etoposide. This change of chemotherapy was warranted due to the results of NWTS-4 which showed that the four-year RFS was considerably improved (27% to 55%) with the addition of cyclophosphamide.

The NWTS- 5 reported four-year RFS of 55.1% and 74.9% for patients with diffuse and focal anaplasia respectively. Four-year RFS estimates for Stage II, III, IV were 82.1%, 68.3% and 37.5% respectively

### Stage V

Synchronous bilateral WT account for 6% of all WT and also pose a special challenge.[3] The goal of therapy for patients with bilateral disease, beyond cure of the tumor, is to spare renal parenchyma to avoid significant renal insufficiency and hence the treatment must be individualized. The NWTS-2 and 3 studies have demonstrated no difference in survival for children who undergo initial bilateral biopsy followed by chemotherapy and then surgical resection compared with patients who have initial resection followed by chemotherapy.[41] However, preoperative chemotherapy often results in significant reduction in tumor size, thereby facilitating subsequent renal salvage. The NWTS-4 reported only 8.2% risk of local relapse following nephron-sparing surgery.[42] The NWTS-5 recommendations for the management of bilateral WT include initial biopsy and local staging followed by chemotherapy (according to abdominal stage and histologic features) and second-look surgery at Week five.[14] Additional radiation and chemotherapy maybe given if indicated but the surgery must be completed by 12 weeks from diagnosis to prevent development of drug-resistant clones.

Initial treatment is with vincristine and dactinomycin if the renal tumors are of favorable histology and not more extensive than Stage II. Those with higher stage and favorable histology disease should receive doxorubicin, vincristine and dactinomycin and those with anaplastic histology should receive cyclophosphamide in addition to vincristine, doxorubicin and etoposide. Following six weeks of chemotherapy, the patient should be reassessed. If serial imaging studies show no further reduction in tumor, a second-look surgical procedure should be performed (partial nephrectomy on one side if possible) if negative margins can be obtained; otherwise, another biopsy should be done to confirm viable tumor.[43] Chemotherapy and/or radiation therapy following the second-look operation is dependent on the response to initial therapy, with more aggressive therapy required for patients with inadequate response to initial therapy observed at the second procedure. Radical nephrectomy is recommended when nephron-sparing surgery is not possible. Approximately 10% of patients with bilateral tumors have anaplastic histology and may benefit from more aggressive chemotherapy and radiation therapy and an aggressive surgical approach at the second-look operation.

Renal transplantation for children with WT is usually delayed until one to two years have passed without evidence of malignancy.[44]

## EXPERIENCE FROM AN INDIAN CENTER

Bhagwat *et al*. (2005) reported their experience from a major oncology tertiary care referral center in India.[45] At their center, Wilms' tumor constitutes 3.5% of approximately 800 new pediatric cancers registered every year and is the most common solid tumor after brain tumors and neuroblastoma. In the earlier years, many of their patients were referred to their institute after primary surgery performed elsewhere, leading to a considerable delay in starting adjuvant treatment. However, in recent times, the majority of patients were operated per primum at their center. Whenever surgery was done outside, every effort was made to stage the disease based on the referring clinician's preoperative and intraoperative examination findings and imaging studies. All patients were evaluated in a multidisciplinary Pediatric Oncology tumor board and treated as per the standard institutional protocol which included three-drug chemotherapy (vincristine, dactinomycin and doxorubicin) for all the patients as given for advanced stages in the NWTS-4 to compensate for lacunae in staging. The overall survival and relapse-free survival of 118 WT patients treated over a 10-year period were 77.6% and 73.4% at 10 years respectively. The overall survivals for Stages I-IV were 83%, 81%, 47% and 75% respectively. These results need to be seen in light of the fact that many patients during this period underwent surgery at an outside center, with incomplete staging, inadequate surgery or tumor spillage during surgery.

## RECURRENT WT

The prognosis of recurrent WT used to be dismal, with the majority of patients receiving the same treatment at salvage as their primary treatment and with survival rates of less than 30% despite standard therapy by current standards (judicious surgery, chemotherapy with or without radiation therapy). However, in recent times, drugs like platinum compounds, ifosphamide, cyclophosphamide, etoposide etc and their combinations have shown considerable activity in patients with relapsed WT. Post-relapse survival rates of 50-60% have been reported with ifosphamide, etoposide and carboplatin (ICE) chemotherapy.[46] Factors which predict better survival are relapse-free interval of more than 12 months, low stage of primary disease, low metastatic burden, two-drug chemotherapy and no previous radiation to tumor bed.[47] Complete resection of the recurrent lesion(s) has also been shown to be a favorable prognostic factor but whether it reflects only low-volume recurrent disease rather than actual survival benefit is yet debatable.[46] High-dose chemotherapy with autologous stem cell rescue is being tried in clinical trials but is presently experimental.[48] Novel drugs and strategies are needed to improve clinical outcome in this group of patients.

Metachronous tumors in the contralateral kidney account for 2% of WT, with children younger than 12 months with perilobar nephrogenic rests at a higher risk.[49] The contralateral tumors need to be treated independently as the tumors in the first kidney. Although patients with metachronous tumors have a worse survival than those with synchronous tumors (five and 10-year OS of 49.1% and 47.2%); in the former group, those developing after 18 months of initial diagnosis fare better than those developing earlier (10-year OS 55.2% vs. 39.6%).[50]

## LONG-TERM SEQUELE

With long-term follow-up data being available in a large number of survivors of WT, the long-term sequele of treatment are becoming better defined. Renal failure after surgical management of unilateral WT is rare. Cardiac problems secondary to anthracycline administration compounded by whole-lung radiotherapy as well as pulmonary complications secondary to whole-lung radiotherapy are real concerns and need to be addressed.[51] Gonadal dysfunction secondary to chemo and/or radiotherapy may occur. Children treated for WT are also at an increased risk of second malignancy, especially if they have also received radiotherapy in addition to chemotherapy.[3]
